# Predictors of hematoma expansion in intracerebral hemorrhage associated with factor Xa inhibitors

**DOI:** 10.3389/fneur.2025.1628563

**Published:** 2025-10-30

**Authors:** Yoji Komatsu, Takao Koiso, Tomokazu Sekine, Kazuki Akutagawa, Hiroki Karita, Norie Kikuchi, Tomosato Yamazaki

**Affiliations:** ^1^Department of Neurosurgery, Hitachi General Hospital, Hitachi, Japan; ^2^Department of Neurosurgery, Institute of Medicine, University of Tsukuba, Tsukuba, Japan; ^3^Department of Neurosurgery, Hitachi Medical Education and Research Center, University of Tsukuba, Hitachi, Japan

**Keywords:** intracerebral hemorrhage, hematoma expansion, direct oral anticoagulant, factor Xa inhibitor, andexanet alfa

## Abstract

**Background:**

Improving the outcomes of patients with intracerebral hemorrhage (ICH) associated with factor Xa inhibitors remains a clinical challenge. Andexanet alfa, a specific reversal agent for factor Xa inhibitors, has the potential to mitigate hematoma expansion (HE). The aim of this study is to identify predictors of HE in ICH associated with factor Xa inhibitor use and to propose appropriate indications for reversal therapy.

**Methods:**

This was a single-center, retrospective observational study that included 68 consecutive patients who developed ICH within 24 h of factor Xa inhibitor intake and were not receiving concomitant antiplatelet therapy. The study period spanned from April 2012 to June 2022. The relationships between HE and patient-related, clinical, hematoma-related, and pharmacological factors were examined.

**Results:**

Hematoma expansion was observed in 18 cases (26.5%) and significantly worsened outcomes (*p* = 0.028). In univariate analysis, significant predictors of HE were an irregular margin and/or heterogeneity of the hematoma on computed tomography (CT) (*p* = 0.009), an initial CT performed within 4 h after drug intake (*p* = 0.034), and edoxaban use (*p* = 0.041). A multivariate analysis identified hematoma morphology on CT (*p* = 0.030) and the initial CT within 4 h after drug intake (*p* = 0.048) as significant predictors. Hematoma volume, interval from onset to initial CT, and coagulation-related laboratory parameters were not significant.

**Conclusion:**

The predictors of HE were an irregular margin or heterogeneous hematoma, and an initial CT performed within 4 h after drug intake. Reversal decisions based on hematoma volume, or interval from onset to initial CT may be inappropriate.

## Introduction

Anticoagulant therapy is strongly recommended for the prevention of cardioembolic stroke in patients with non-valvular atrial fibrillation (1). However, intracerebral hemorrhage (ICH) remains one of the major adverse events associated with anticoagulant use. Compared with vitamin K antagonists (VKAs), direct oral anticoagulants (DOACs) reduced the incidence of ICH by 48% (2). Furthermore, meta-analyses demonstrated that even when ICH occurred, patients receiving DOACs were more likely to have smaller hematoma volumes and to experience lower rates of hematoma expansion (HE) (3). Larger hematoma volumes and HE have been associated with worse clinical outcomes (4).

Several factors have been identified as predictors of HE in patients with spontaneous ICH, including the use of antiplatelet or anticoagulant agents, a shorter interval from symptom onset to initial computed tomography (CT) scanning, a larger baseline hematoma volume, an irregular margin and/or heterogeneity of the hematoma on non-contrast CT, and the presence of contrast extravasation within the hematoma on contrast-enhanced CT (5–11). While anticoagulant use itself is recognized as a risk factor for HE, specific predictors of HE among patients receiving anticoagulation therapy have not yet been fully characterized. Moreover, it remains unclear whether the predictive factors established in the general ICH population are directly applicable to those receiving anticoagulant therapy.

In patients receiving VKAs, a prothrombin time-international normalized ratio (PT-INR) ≥ 2.0 has been identified as a risk factor for HE (12). In contrast, predictive factors for HE in patients taking DOACs remain unclear. DOACs are classified as thrombin inhibitors and factor Xa inhibitors based on their pharmacological targets. Regarding dabigatran, a thrombin inhibitor, the activated partial thromboplastin time (APTT) has been suggested to partially reflect anticoagulant activity (13–15). However, conventional coagulation assays have proven to be poor indicators of the pharmacological activity of factor Xa inhibitors (13–15).

Reversal agents for oral anticoagulants have been developed, and the interruption and reversal of anticoagulation are both recommended in the setting of ICH (1). Nevertheless, reversal therapy poses a clinical dilemma because it may increase the risk of thromboembolic complications. Andexanet alfa, a specific reversal agent for factor Xa inhibitors, demonstrated excellent or good hemostatic efficacy in 80% of cases when administered within 18 h after the last anticoagulant dose; however, ischemic stroke occurred in 4.6% of patients within 30 days following reversal (16–18).

The identification of reliable predictors of HE in patients treated with factor Xa inhibitors is essential for optimizing treatment strategies in anticoagulant-associated ICH. Therefore, the present clinical study aimed to clarify the incidence of HE, its effects on clinical outcomes, and predictors of HE in patients with spontaneous ICH who were receiving factor Xa inhibitors.

## Materials and methods

### Study design, and participants

This was a retrospective, single-center observational study that used data from medical records. This study included consecutive cases of ICH that occurred between April 2012, when factor Xa inhibitors were first introduced in Japan, and June 2022, when the reversal agent andexanet alfa became clinically available.

Inclusion criteria were as follows: patients who had been regularly taking one of the factor Xa inhibitors (rivaroxaban, apixaban, or edoxaban) for at least 1 month prior to the onset of hypertensive ICH, who developed ICH within 24 h of taking a factor Xa inhibitor and presented to Hitachi General Hospital within 24 h of their onset.

Exclusion criteria included cases with the concomitant use of antiplatelet agents, cases in which the primary lesion was intraventricular hemorrhage, and cases lacking follow-up CT imaging. Additionally, patients with vascular abnormalities capable of causing hemorrhage, such as aneurysms, arteriovenous malformations, or Moyamoya disease, were excluded.

### Outcomes

Clinical outcomes were evaluated using the modified Rankin Scale (mRS) 3 months after the onset of ICH, with favorable outcomes defined as an mRS scores of 0–3.

Hematoma volumes were calculated using the ellipsoid formula: (length × width × height)/2, based on measurements obtained from CT images with a slice thickness of 2.5 mm (19).

HE was defined as an increase in hematoma volume of ≥33% or an absolute increase of ≥6 mL within 24 h of the initial CT scan (20).

### Image assessment and hematoma morphological categories

The initial CT was, in principle, obtained within 30 min of hospital arrival.

Follow-up CT scans were generally obtained at 3, 12, and 24 h after the initial CT. Additional imaging was conducted if clinical deterioration was observed.

**Figure 1:**
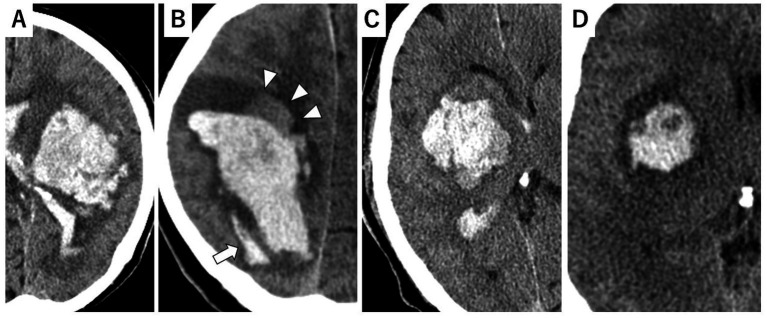
Representative examples of CT markers. **(A)** Island sign. **(B)** Satellite sign (allow) with blend sign (allow heads). **(C)** Blend sign. **(D)** Black hole sign.

The CT morphology of the hematoma was assessed based on its margin configuration and Hounsfield unit (HU) characteristics. An irregular hematoma margin was defined as meeting at least one of the criteria for the island sign or the satellite sign. Heterogeneous hematoma density was defined as meeting at least one of the criteria for the blend sign or the black hole sign. The following imaging signs were defined as follows (7, 9–11):

Island sign: Defined as three or more small hematomas scattered apart from the main hematoma, or more than four small hematomas partially or completely connected to the main hematoma (Figure 1A).
Satellite sign: Defined as high-density lesions observed around the hematoma (Figure 1B).
Blend sign: Defined as a hyperattenuating area blends with an adjacent relatively hypoattenuating region, with a well-defined margin (Figure 1C).
Black hole sign: Defined as a relatively hypoattenuated round, oval, or rod-shaped area within a hyperattenuated hematoma (Figure 1D).

### Dose of factor Xa inhibitor

Factor Xa inhibitors are administered in both standard and reduced dosing regimens, with specific dose reduction criteria defined for each agent based on creatinine clearance (CrCL) (21), age, body weight, and concomitant medications. In Japan, the approved standard and reduced daily doses of rivaroxaban are 15 mg and 10 mg once daily, respectively, which differ from the internationally recommended dosing guidelines. In contrast, the dosing regimens for apixaban and edoxaban in Japan align with global standards, with standard and reduced daily doses of 10 mg (5 mg twice daily) and 5 mg (2.5 mg twice daily) for apixaban, and 60 mg and 30 mg once daily for edoxaban, respectively.

Overdosing was defined as the administration of a standard dose in patients who met the criteria for dose reduction.

### Factors analyzed

The following factors were analyzed for their relationship with HE:

Patient-related factors: Age, sex, hypertension (HT), hyperlipidemia (HL), diabetes mellitus (DM), alcohol intake >20 g ethanol per day (22), and current smoking.
Clinical factors: systolic blood pressure (sBP) on arrival, sBP after 3 h, CrCL, the Child-Pugh classification (23), platelet count, PT-INR, and APTT.
Hematoma-related factors: Location, volume on baseline CT, interval from onset to initial CT, and CT morphology.
Pharmacological factors: Interval from last intake of factor Xa inhibitor to ICH onset, interval from last intake to initial CT, the type of factor Xa inhibitor, dosage, appropriateness of the dose compared with the recommended regimen.

### Statistical analysis

Statistical analyses were performed using the median test for continuous variables and the chi-squared test or Fisher’s exact test for categorical variables, with a significance threshold set at *p* ≤ 0.05. Variables with a *p*-value ≤0.20 in the univariate analysis were entered into a multivariate logistic regression model. All statistical analyses were conducted using Stat Mate version 4.01 (ATMS Co., Ltd., Japan).

## Results

### Patient characteristics

Ninety-two cases of spontaneous ICH occurring within 24 h of factor Xa inhibitor intake were identified during the study period. Of these, 24 cases were excluded for the following reasons: two in which intraventricular hemorrhage was the primary lesion, 12 cases involving the concomitant use of antiplatelet agents, and 10 cases lacking follow-up CT imaging. Among the latter, five patients underwent surgical intervention after initial CT, and five were in critical condition and unable to undergo repeat imaging.

We included 68 patients in this study. The median age was 77.5 years (IQR 70.8–85.0), and 39 (57.4%) were male. The factor Xa inhibitors and dosages were rivaroxaban in 32 patients (standard dose, 14; reduced dose, 18), apixaban in 15 (standard dose, 7; reduced dose, 8,), and edoxaban in 21 (standard dose, 8; reduced dose, 13). The indications for factor Xa inhibitor therapy were non-valvular atrial fibrillation, including paroxysmal cases, in 62 patients; deep vein thrombosis or pulmonary embolism in 14; and both conditions in 8.

### Outcomes

The HE was observed in 18 of the 68 included cases (26.5%). Among these, 10 cases occurred within 6 h and 7 within 12 h after the initial CT. In one case, hematoma enlargement was confirmed at the 24-h evaluation.

**Figure 2:**
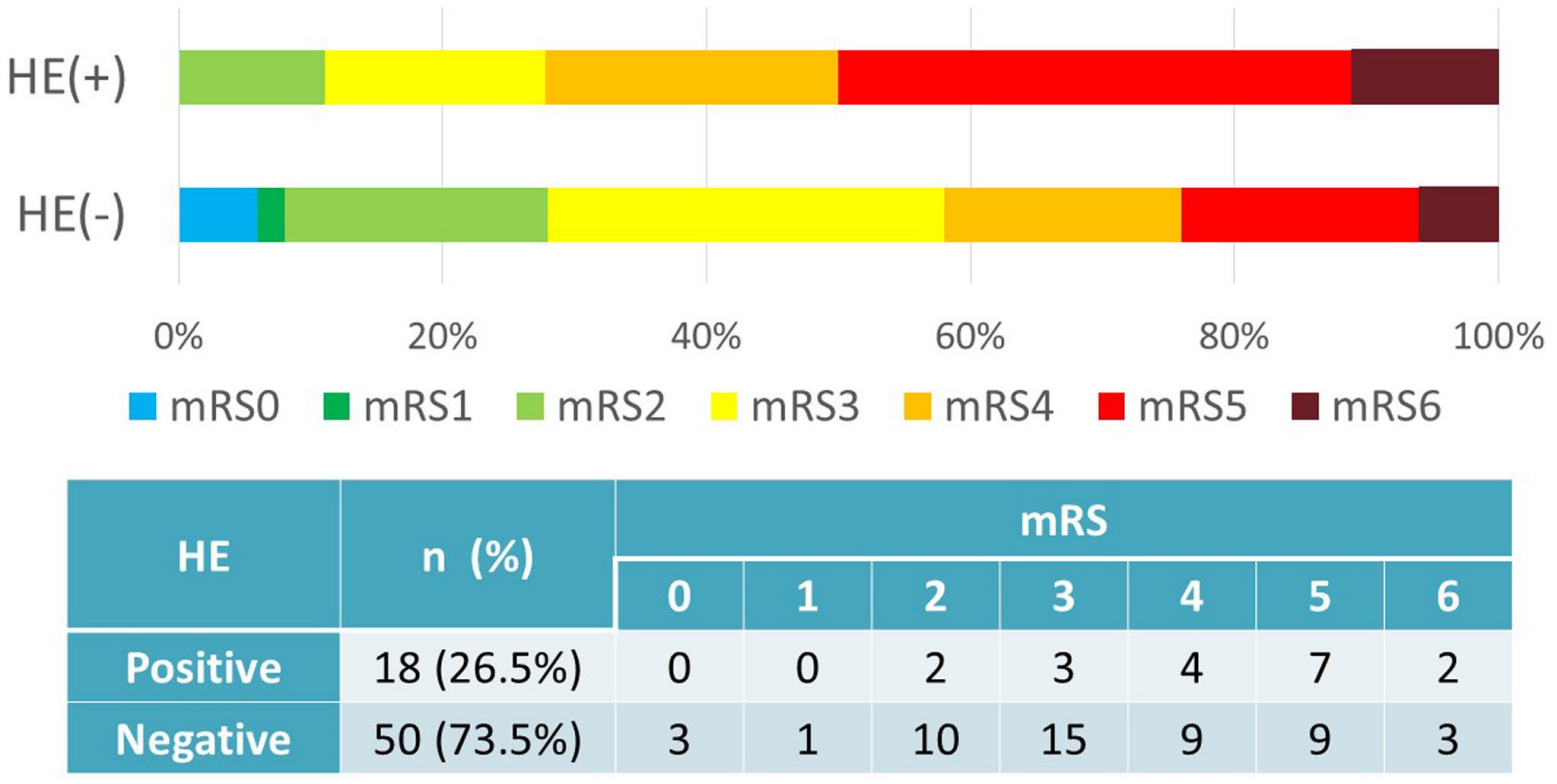
Hematoma expansion vs. outcomes (mRS). Favorable outcomes (mRS0–3) were achieved in 29 cases (58.0%) without HE and in 5 cases (27.8%) with HE. HE correlated with worse functional outcomes (*p* = 0.028, *χ*^2^ test). HE, hematoma expansion; mRS, modified Rankin Scale.

Favorable outcomes at 3 months were achieved in 29 cases (58.0%) without HE and in 5 cases (27.8%) with HE. The presence of HE was significantly associated with worse functional outcomes (*p* = 0.028, *χ*^2^ test) (Figure 2).

During the period of factor Xa inhibitor discontinuation following ICH onset, thromboembolic events occurred in 3 patients (4.4%), comprising one case each of pulmonary embolism, myocardial infarction, and cerebral venous sinus thrombosis. The outcomes were fatal in the pulmonary embolism and myocardial infarction cases, whereas the patient with cerebral venous sinus thrombosis had a mRS score of 4.

### Predictors of HE

The median values or incidence of various patient, clinical, hematoma, and drug factors stratified by the presence or absence of HE are summarized in Table 1.

**Table 1:** Univariate and multivariate analyses of the involvement of each factor in hematoma expansion.

Variable		HE (+)	HE (−)	Univariate	Multivariate	
		*n* = 18	*n* = 50	*p*-value	OR (95%CI)	*P*-value
Patient-related factors						
Sex	Male	72.2%	52.0%	0.226		
Age, years	Median (IQR)	73.5 (68.3–84.8)	80 (72–85)	0.410		
HT	Yes	83.3%	86.0%	0.909		
HL	Yes	16.7%	18.0%	0.816		
DM	Yes	22.2%	16.0%	0.816		
Alcohol	>20 g per day	27.8%	14.0%	0.340		
Smoking	Current	16.7%	6.0%	0.377		
Clinical factors						
sBP on arrival, mmHg	Median (IQR)	190 (162–201)	172 (154–192)	0.066	1.01 (0.99–1.02)	0.245
sBP after 3 h, mmHg	Median (IQR)	124 (118–130)	126 (119–133)	0.823		
CrCL, mL/min	≦50	33.3%	44.0%	0.430		
Child-Pugh classification	B or C	5.6%	4.0%	0.694		
Platelets, ×10^4^/cmm	Median (IQR)	18.6 (14.4–21.2)	19.2 (15.0–22.0)	0.169	0.98 (0.89–1.09)	0.766
Pt-INR	Median (IQR)	1.04 (1.01–1.13)	1.10 (1.04–1.18)	0.219		
APTT, seconds	Median (IQR)	32.9 (28.8–35.9)	31.3 (29.0–34.0)	0.987		
Hematoma-related factors						
Location	Lobar	11.1%	20.0%	0.626		
Volume on baseline CT, mL	Median (IQR)	12.2 (3.9–36.2)	5.8 (2.4–16.4)	0.054	0.99(0.95–1.03)	0.737
Interval from onset to CT, hours	Median (IQR)	2.0 (0.5–4.5)	2.0 (1.0–5.0)	0.720		
Irregularity and/or heterogeneity	Yes	77.8%	38.0%	0.009	4.49(1.14–18.1)	0.030
Pharmacological factors						
Interval from last dose to onset, hours	≦4	50.0%	32.0%	0.283		
Interval from last dose to CT, hours	≦4	44.4%	16.0%	0.034	3.75(1.01–13.9)	0.048
Type of factor Xa inhibitor	Rivaroxaban	27.8%	54.0%	0.041	2.01 (0.64–4.46)	0.200
	Apixaban	22.2%	22.0%			
	Edoxaban	50.0%	24.0%			
Dosage	Standard dose*	38.9%	44.0%	0.707		
Comparison of optimal doses	Over dose	18.8%	5.0%	0.113	5.68 (0.64–43.3)	0.089

^*^standard dose: rivaroxaban 15 mg once a day, apixaban 5 mg twice a day, edoxaban 60 mg once a day.

HT, hypertension; HL, hyperlipidemia; DM, diabetes mellitus; sBP, systolic blood pressure; CrCL, creatinine clearance; PT-INR, prothrombin time-international normalized ratio; APTT, active partial thrombin time; CT, computed tomography; HE, hematoma expansion; IQR, interquartile range.

In univariate analysis, significant predictors of HE were hematoma morphology on CT (*p* = 0.009), an interval of ≤4 h from the last factor Xa inhibitor intake to the initial CT (*p* = 0.034), and edoxaban use (*p* = 0.041). Factors showing a trend toward association included larger initial hematoma volume (*p* = 0.054), higher sBP on admission (*p* = 0.066), factor Xa inhibitor overdose (*p* = 0.113), and lower platelet count (*p* = 0.169). In contrast, HT, HL, DM, Child–Pugh classification, sBP at 3 h, PT-INR, APTT, an interval of ≤4 h from the last factor Xa inhibitor intake to symptom onset, interval from symptom onset to initial CT, and factor Xa inhibitor dosage were not associated with HE.

Multivariate analysis of variables with *p* ≤ 0.20 in univariate analysis identified irregular margin and/or heterogeneity of the hematoma on non-contrast CT (*p* = 0.030) and an interval of ≤4 h from the last Xa inhibitor intake to the initial CT (*p* = 0.048) as independent predictors of HE. Factor Xa inhibitor overdose demonstrated a trend toward association with HE (*p* = 0.089), whereas the type of Xa inhibitor (*p* = 0.200), sBP on admission (*p* = 0.245), initial hematoma volume (*p* = 0.737), and platelet count (*p* = 0.766) were not predictive.

## Discussion

In this clinical study, HE occurred in 26.5% of patients with spontaneous ICH who were taking factor Xa inhibitors without concomitant antiplatelet therapy. HE was associated with worse clinical outcomes, as measured by a mRS score of 4–6. Significant predictors for HE were the presence of an irregular margin and/or heterogeneity of the hematoma on non-contrast CT, and an interval ≤4 h from the last Xa inhibitor intake to initial CT. Notably, factors that are commonly reported as predictors of HE in general ICH, such as the interval from symptom onset to initial CT and baseline hematoma volume, were not significant in this cohort.

These findings suggest that predictive indicators of HE differ in patients taking factor Xa inhibitors, which may be due to the distinct pharmacological effects of these agents on coagulation.

### Hematoma morphology

The presence of an irregular margin and/or heterogeneity of the hematoma on non-contrast CT associated with HE. These imaging features have been recognized in previous studies as predictors of HE in general ICH (5–11).

Heterogeneous attenuation on CT is considered to reflect incompletely clotted blood, indicating ongoing bleeding.

Areas within a hematoma with attenuation values below 30 Hounsfield units (HU) have been linked to a higher risk of HE. Fully clotted blood typically demonstrates values of approximately 100 HU, while partially clotted or active bleeding regions range from 30 to 50 HU (24). Irregular hematoma margins may indicate continued bleeding from ruptured perforating arteries at the hematoma edge.

Despite the predictive value of these features, HE still occurred in 22.2% of cases without these CT features. Blacquiere et al. (6) reported that density heterogeneity demonstrated a sensitivity of 44% and a specificity of 69%, whereas margin irregularity showed a sensitivity of 72% and a specificity of 46%. In contrast, Kim et al. (10) found that 38.8% of hematomas with density heterogeneity did not exhibit expansion, and 41.4% of those with margin irregularity also did not demonstrate growth. It should be noted that the risk of HE cannot be ruled out even in cases that do not exhibit these hematoma morphologies.

The “target sign” on contrast-enhanced CT is another established indicator of active bleeding; however, we were unable to assess this sign in the present study due to the limited number of patients who underwent contrast-enhanced imaging (11). Future studies incorporating this parameter may improve the prediction of HE.

### Interval from factor Xa inhibitor intake to initial CT

In general ICH, a shorter interval from symptom onset to CT is associated with an increased risk of HE, which may be due to incomplete hemostasis early in the hemorrhagic process (8).

In the present study, we examined both the interval from factor Xa inhibitor intake to initial CT and the interval from symptom onset, only the former was significantly associated with HE.

Factor Xa inhibitors reach peak activity within 1–4 h of their administration and have varying half-lives: rivaroxaban (5–9 h), apixaban (10–14 h), and edoxaban (9–11 h) (15, 25–27). These pharmacological characteristics indicate that anticoagulant activity is higher within 4 h after administration. OD further increases the plasma concentration of factor Xa inhibitors. In addition, the longer half-life of edoxaban compared with other agents may explain its association with HE observed in the univariate analysis. Therefore, in order to predict HE in patients receiving factor Xa inhibitors, it would be ideal to evaluate the degree of anticoagulant activity in each patient. However, conventional coagulation assays have proven to be poor indicators of the pharmacological activity of factor Xa inhibitors (13–15).

While HE was identified as high-risk within 4 h of the last intake, 55.6% of HE cases occurred beyond this timeframe. Accordingly, it would be inappropriate to consider patients ineligible for reversal therapy solely on the basis that more than 4 h have elapsed since the last intake.

Godier et al. (14) reported that the anticoagulant effects of DOACs may persist for up to 72 h in patients with moderate renal impairment. Routine coagulation assays are insufficient to reliably quantify this effect. Due to the potential for ongoing anticoagulant activity, reversal therapy should be considered for all ICH cases occurring within 24 h of the last factor Xa inhibitor dose.

The benefits of reversal beyond the 24-h window remain unclear and, thus, warrant further investigation.

### Baseline hematoma volume

In general ICH, larger hematoma volumes have been associated with an increased risk of HE, which may be attributed incomplete hemostasis of the perforating artery leading to hematoma enlargement and mechanical stress at the hematoma margins promoting additional vascular rupture (5).

In the absence of anticoagulant effects, small hematomas typically do not enlarge because hemostasis is already complete. In contrast, in patients receiving factor Xa inhibitors, the suppression of coagulation increases the risk of both persistent bleeding and new vessel rupture, even in cases with smaller hematomas.

The interval from the last dose to ICH onset was not a predictor of HE. A possible explanation is that HE occurring prior to the initial CT could not be assessed in this study. It should be acknowledged that the occurrence of HE during the early phase of ICH may result in a large hematoma by the time of hospital arrival.

Although smaller hematomas are generally associated with better outcomes, the present results indicate that hematoma volume was not a significant predictor of expansion in this population. Therefore, even small hematomas in patients treated with factor Xa inhibitors warrant consideration of reversal therapy to prevent expansion and improve outcomes.

### Indications for the reversal of factor Xa inhibitors

Delcourt et al. (4) reported that a hematoma volume increase of ≥6 mL or ≥33% significantly worsened outcomes (mRS 3–6), with adjusted odds ratios of 1.72 and 1.67, respectively. Consistent with this finding, the present study demonstrated that HE was associated with poor functional outcomes in patients taking factor Xa inhibitors.

Andexanet alfa achieves rapid and effective reversal of factor Xa inhibitors; however, the optimal criteria for its administration have yet to be defined (16).

The predictors of HE identified in this study were an irregular or heterogeneous hematoma morphology and ICH onset within 4 h after factor Xa inhibitor intake. The former likely reflects incomplete hemostasis of the hematoma, while the latter indicates high factor Xa inhibitor activity. In the presence of these factors, reversal of factor Xa inhibitors should be considered. However, it must be recognized that HE may occur even in the absence of these predictors, and the indication for reversal should therefore be carefully considered. By contrast, basing reversal decisions on hematoma volume or the interval from ICH onset—both of which have been reported as predictors of HE in general ICH—is not appropriate.

Thromboembolic complications were observed in 4.4% of patients during anticoagulation therapy interruption due to ICH in this study. Andexanet alfa may reduce the duration of treatment interruption. Whether a shortened discontinuation period of factor Xa inhibitors can effectively prevent thromboembolic events requires further investigation.

### Limitation

The limitations of this study include its retrospective design and the fact that it was conducted at a single center. Future research involving larger, multicenter clinical studies is warranted.

## Conclusion

HE occurred in 26.7% of patients with spontaneous ICH taking factor Xa inhibitors and was associated with unfavorable clinical outcomes.

Among patients who had taken factor Xa inhibitors within 24 h, irregular or heterogeneous hematoma morphology and an interval of ≤4 h from the last factor Xa inhibitor intake to the initial CT were significant predictors of HE. Decisions regarding reversal based on hematoma volume or the interval from onset to initial CT may therefore be inappropriate.

## Data Availability

The raw data supporting the conclusions of this article will be made available by the authors, without undue reservation.
